# Human Monocytes Plasticity in Neurodegeneration

**DOI:** 10.3390/biomedicines9070717

**Published:** 2021-06-23

**Authors:** Ilenia Savinetti, Angela Papagna, Maria Foti

**Affiliations:** School of Medicine and Surgery, University of Milano-Bicocca, 20900 Monza, Italy; i.savinetti@campus.unimib.it (I.S.); angela.papagna@unimib.it (A.P.)

**Keywords:** neurodegeneration, innate immunity, human monocytes, trained immunity, epigenetics, single cells analysis, gene expression

## Abstract

Monocytes play a crucial role in immunity and tissue homeostasis. They constitute the first line of defense during the inflammatory process, playing a role in the pathogenesis and progression of diseases, making them an attractive therapeutic target. They are heterogeneous in morphology and surface marker expression, which suggest different molecular and physiological properties. Recent evidences have demonstrated their ability to enter the brain, and, as a consequence, their hypothetical role in different neurodegenerative diseases. In this review, we will discuss the current knowledge about the correlation between monocyte dysregulation in the brain and/or in the periphery and neurological diseases in humans. Here we will focus on the most common neurodegenerative disorders, such as Alzheimer’s disease, Parkinson’s disease, amyotrophic lateral sclerosis and multiple sclerosis.

## 1. Introduction

Neurodegeneration is an age- and disease-related process, characterized by the progressive loss and dysfunction of CNS neurons and structures.

Aging is a physiological condition of neuronal damage over time, and distinguishing neurodegeneration patterns from normal aging or related diseases poses a clear challenge [1,2]. Indeed, neurodegeneration is known to be directly mediated by cellular aging [3].

**Figure 1:**
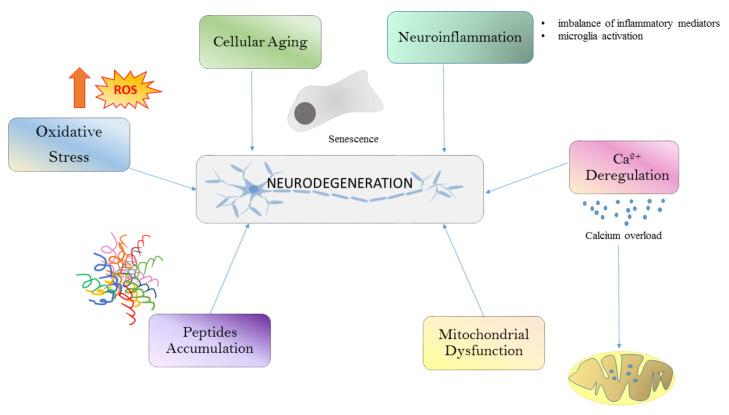
Common pathways that lead to neurodegeneration. Neuronal damage in neurodegenerative diseases is induced by ROS generation, cellular aging, neuroinflammation, Ca^2+^ dysregulation, mitochondrial dysfunction and peptide accumulation. ROS, reactive oxygen species.

Different conditions such as oxidative stress (OS), calcium deregulation, neuroinflammation, and mitochondrial dysfunction and aggregation are all well-known drivers of neurodegeneration (Figure 1). All these processes are linked together in a long cascade of intracellular events. The oxidative stress determines mitochondrial dysfunctions at respiratory chain levels, increases cytosolic calcium, and plays a role in protein aggregation. The initial aggregation observed in Alzheimer’s disease (AD) could be a way to protect the microenvironment from oxidative damage. In fact, OS induces macroautophagy of Aβ aggregates [4]. Although defects in neurons and glia may explain this degeneration, changes in the systemic peripheral immune system can also be involved in age-related brain dysfunction [5].

In recent years, the dynamic role of the blood–brain barrier (BBB) mediating peripheral cell migration into the brain has emerged, reflecting the contribution of peripheral systemic factors to different neurodegenerative aspects. For example, BBB damage has been observed during normal aging and becomes exaggerated in cases of cognitive impairment, regardless of the Aβ or Tau pathology [6].

Nevertheless, much remains to be clarified, and a lot of questions still remain unanswered: (i) Is neurodegeneration the consequence of neurological diseases, or are neurological diseases the consequence of neurodegeneration? (ii) To what extent does aging or specific disease impact the neurodegenerative process?

## 2. Monocytes: Different Subtypes for Different Functional Roles

Monocytes are mononuclear cells that develop in the bone marrow from a myeloid progenitor, and circulate within the bloodstream. In response to particular stimuli (e.g., infection) monocytes migrate into tissues and differentiate into macrophages (Mϕ) or dendritic cells (DC) to eliminate the pathogens by phagocytosis, cytokine production, and antigen presentation. In the blood, monocytes can be divided into different subsets, based on the expression of the surface markers CD14 and CD16 [7]. Until now, at least three subsets have been described: the so called “classical”, “intermediate”, and “non-classical” monocytes. Classical monocytes represent 85–90% of the total monocytes; they are characterized by high CD14 expression but lack CD16 (CD14^++^/CD16^−^). Intermediate and non-classical monocytes constitute the remaining 10–15% and are characterized by high CD14/low CD16 expression (CD14^++^/CD16^+^) and high CD16/lower CD14 expression (CD14^+^/CD16^++^), respectively [8,9,10]. In recent years, various studies have been conducted to characterize the different subsets. Intermediate monocytes have been shown to express significantly higher levels of Toll-like receptors (TLRs) 2, 4, and 5 as compared to the other two subsets, indicating a primarily pro-inflammatory function [11]. Additionally, intermediate monocytes express high levels of CD80, CD86, and HLA-DR suggesting also a role in antigen presentation. However, the non-classical monocytes were also found to express high levels of CD80 and CD86, indicating an antigen-presenting capability also for this subset. Interestingly, the classical monocytes express low levels of TLRs and co-stimulatory molecules and higher levels of CD36 and CD163, suggesting that the majority of blood monocytes are primarily phagocytic in nature [11] (Table 1).

Since the distinction and function of monocyte subsets differ among studies, questions regarding different functional contribution of monocyte subsets are still debatable. One way to address this issue is to study subset gene expression profiles. Microarray technology has been extensively applied to study monocytes to discover if specific gene expression profiles exist for each human subset [12,13,14]. Recently, a complete compendium of monocyte gene expression studies has been published by collecting 93 public datasets corresponding to 4516 transcriptomes. The analysis included mainly human monocytes purified by healthy controls (58 subjects) and monocytes covering autoimmunity, infections, cancer, and cardiovascular and kidney diseases (35 subjects) [15]. Some of these studies address the molecular signature in monocyte subsets. In classical monocytes, a significant enrichment in angiogenesis, tissue repair function, and response to stimuli, including responses to bacterial components, toxins, and hormones were described [16]. They promote antimicrobial activity through upregulation of myeloperoxidase (MPO), lysozyme C precursor (LYZ), S100 calcium binding protein A9 (S100A9), eosinophil cationic protein precursor (RNase3), phospholipase B domain containing 1 (PLBD1), and Cathepsin G (CTSG) at both mRNA and protein levels [17]. The data suggested that classical monocytes display high plasticity, being capable of responding to diverse stimuli. Moreover, classical monocytes showed elevated levels of several genes involved in carbohydrate metabolism, including a major regulator of the glycolytic pathway hypoxia-inducible-factor 1-alpha (HIF-1A) [18]. In intermediate monocytes, significant enrichment for genes under major histocompatibility complex (MHC) class II processing and presentation were identified, suggesting a prominent role in antigen presentation function [16]. Finally, the non-classical monocytes subset expressed several genes involved in cytoskeleton rearrangement. Indeed, they exhibit an upregulation of activation-induced cytidine deaminase (AICDA) and apolipoprotein B mRNA-editing enzyme catalytic subunit 3A (APOBEC 3A), which codify proteins that phosphorylate the immunoreceptor tyrosine-based activation motif (ITAM) of Fc receptors leading to recruitment of downstream genes necessary for cytoskeletal remodeling [19]. These findings may explain the molecular basis of their highly motile behavior observed in vivo [8]. Moreover, non-classical monocytes display higher transcriptional activity of genes encoding components of the mitochondrial respiratory chain [18].

All the above transcriptomics data represent an excellent collection of datasets that could be used in the future to build specific monocytes subset classifiers able to predict subset specific phenotypes under diverse in vitro and in vivo experimental settings [20,21,22].

Besides microarray studies, high throughput sequencing methods such as next generation sequencing (NGS) have become available, and data are now generated by using them [23,24,25,26]. The so-called RNAseq has been applied to dissect the transcriptomes of different cell types in both health and disease context [27]. Moreover, a further development of the technology is represented by RNAseq at the single cell level, which is the most recent major achievement in transcriptomics analysis. Single cell analysis promises to finally dissect the subset subdivisions within the immune cell subpopulations [28]. Blood monocyte scRNAseq indicated that classical and non-classical monocytes belong to two major transcriptionally defined clusters [29]. The data suggested that a high proportion of intermediate monocytes belong to either of the two groups. A subgroup of cells within the non-classical monocytes additionally formed two distinct clusters suggesting that intermediate monocytes may consist of multiple known and unknown populations of cells [29]. It can be concluded that the three subsets have been generally confirmed by the molecular analysis; however, the exact similarity between the intermediate monocytes and the other two subsets is still a matter of debate and probably their functional activity depends on the cellular microenvironment in which the cells operate within each tissue.

The monocyte plasticity and complexity is further highlighted during the disease process [30]. In fact, gene expression during different types of diseases has identified several molecularly distinct monocyte cellular subsets. Again, gene expression has paved the way to understand this diversity. Studies in atherosclerosis and in infectious diseases have described changes in monocyte genetic signatures before and after disease [23,31]. Moreover, in some cases, disease severity was associated to monocyte gene expression activation suggesting that monocyte activity may be associated with disease progression [32]. The presence of a specific monocyte population in severe COVID19 disease again suggests that monocyte plasticity may be influenced by a variety of factors in the tissue microenvironment during environmental perturbation [33]. A similar finding was described during Toxoplasma infection in which a specific monocyte subset appeared compared to uninfected cells [23]. Finally, on the same line, a recent study showed that different subsets are generated during acute or chronic phase of brain disease during neuroinflammation [34], again substantiating the hypothesis that monocytes can locally differentiate from one subset to another depending on tissue-specific signals. Therefore, we can conclude that tissue complexity and genetic reprogramming may explain the extraordinary plasticity of this cell type.

## 3. Epigenome Regulation of Monocytes Plasticity in Neurodegeneration

Monocytes are characterized by a remarkable degree of plasticity and ability to rapidly adapt to a wide range of microenvironments [35]. A number of studies have demonstrated the importance of epigenetics in the regulation of monocyte phenotypes [36]. Epigenetic modifications are influenced by diverse factors able to induce cell-specific changes to the environmental exposure. Since monocytes circulate in the blood, and their epigenome maybe influenced by the presence of diverse molecules such as food-derived metabolites, and in case of pathological conditions also by different inflammatory mediators. So, beside their expression profiles, the definition of the epigenetic state of monocytes is essential to understand their role in health and disease. Epigenetics refers to modifications that do not alter the DNA sequence but instead control how information encoded in DNA is expressed and regulated in a tissue- and context-specific manner. To date epigenetic changes, include the following categories: (i) DNA methylation, (ii) histone modifications and (iii) non-coding RNA.

In general, DNA methylation is associated with transcriptional repression and is related to the transfer of a methyl group to the cytosine base of the DNA by DNA methyltransferases (DNMTs) to form 5-methyl-citosine (5 mC). Histone modifications regulate cellular phenotypes by adding or removing the acetyl or methyl group in histone proteins; these activities are regulated by acetyltransferases (HATs) and histone deacetylases (HDACs), respectively. Histone acetylation is linked to transcriptional activity whereas histone deacetylation is associated with transcriptional repression [37]. Similarly, methylation and demethylation of histones is achieved by histone methyltransferases (HMTs) and histone demethylases (HDMs), respectively. Histone methylation can induce both transcriptional activation and transcriptional repression, depending on the number and location of the methyl groups.

Epigenetic changes have also been implicated in the pathogenesis of a number of neurodegenerative diseases, such as Alzheimer’s, Parkinson’s, or amyotrophic lateral sclerosis (ALS) [38,39,40]; nevertheless, a detailed role of monocyte epigenetics in these diseases is still missing. Regarding multiple sclerosis (MS), to date, small number of studies have addressed the role of epigenetically mediated changes in blood of MS patients. Methylation profiles of mainly CD4^+^, CD8^+^ T cells, B cells, monocytes, and cell-free plasma DNA were reported, and the most interesting findings were related to hypomethylation on the IL17A promoter region, which is known to correlate with Th17 cell lineage generation and a decrease in the methylation pattern located in the HLA-DRB1 gene suggesting that the DRB1 haplotype may influence the association observed between the methylation level at DRB1 CpGs and MS risk [41,42,43].

Monocyte epigenomics was described in one study [44]. The authors found that B cells and monocyte methylation profiles were the most different between relapsing remitting multiple sclerosis (RRMS) and healthy controls. No significant differences were described for CD4 and CD8 T cells.

Usually, non-coding RNA (ncRNA) are grouped under the epigenetic mechanism as they have an important role in regulating coding and non-coding regions of the genome beside a direct regulation of the gene expression. There are several subtypes of long and short ncRNA species, many of which are involved in regulation of gene expression, and can be further grouped according to their genomic origins and biogenic processes. The best studied of short ncRNAs are the microRNAs which are 20–23 nucleotides (nts) in length and usually recognize target mRNAs by complementarity to seed region in the 3′-UTR of the genes. MicroRNAs profiling in MS has also been extensively studied in peripheral blood mononuclear cells, whole blood, lymphocytes, and cell-free plasma to elucidate their role in MS pathogenesis. Although promising, the results obtained were highly controversial, probably because of the heterogeneity of the cohort of patients selected, the different clinical stages and the different types of samples analyzed. All these data strongly suggest the need to define strategies for the development of a precision medicine approach so that genomics and/or epigenomics analysis will help to define the precise pathogenic mechanisms operating within a subgroup of well-defined MS patients’ clinical stage.

Finally, microRNA can also be released into membrane-bound vesicles (also referred to as extracellular vesicles, or EVs) and several studies have reported a role of EVs in neurodegeneration. Most of the studies examined miRNAs and RNAs in EVs isolated from cultured cell media from the CNS cells (e.g., neurons, astrocytes, microglia, and oligodendrocytes), only few in the plasma of PD, AD, and ALS [45,46,47,48,49,50]. It was suggested that monocytes plasticity can also be modulated by microRNA molecules that are present within EVs. Indeed, in vitro experiments showed that endothelial-derived EVs promoted monocytes activation by enhancing monocytes migration through an endothelial monolayer [51]. In addition, a recent study also showed a reduction of monocyte-derived EVs in samples obtained from patients after one year of fingolimod treatment suggesting that EVs were indeed implicated through the modulation of monocyte activity with the mechanisms of action of immunomodulatory treatments [52,53].

In summary, evidence suggests that epigenetics play a role in monocyte phenotypes. Thus, it will be important to understand the type of mechanisms that drive monocyte diversity and plasticity in the context of neurodegeneration. Dysregulated epigenetic changes may contribute to the persistence of the disease, and therefore, a future challenge will be to understand how to modulate these modifications to develop novel treatments for neurodegenerative diseases.

## 4. Trained Immunity: A New Role for Monocytes?

**Figure 2:**
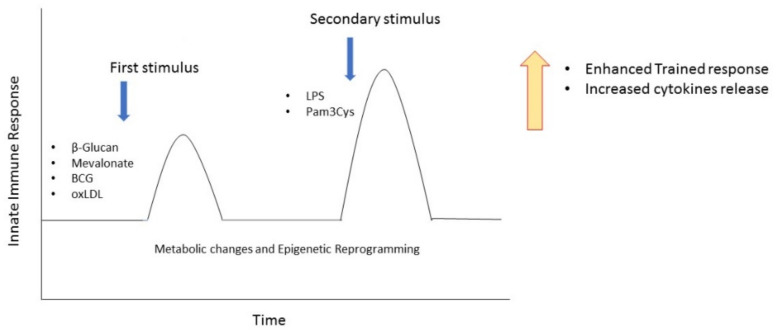
Induction of trained immunity in monocytes. Repeated stimulation of monocytes with β-Glucan, Mevalonate, BCG, or oxLDL determines an enhanced proinflammatory response after a secondary stimulus. Arrows represent the moment of the first and the second stimulation. BCG, bacillus Calmette–Guérin; oxLDL, oxidized low-density lipoprotein.

In the last few years, a new concept of immunological memory on innate immune cells has emerged. This process was named trained immunity (TI) [54]: monocytes exposed to a primary stimulus, such as β-glucan, bacillus Calmette–Guérin (BCG) vaccine, oxidized low-density lipoprotein (oxLDL) [55], or mevalonate [56], and then exposed to a secondary stimulus which can be either an infection or a vaccine, increase the magnitude of the pro-inflammatory response. The secondary stimulus can be completely different from the first one, suggesting that monocytes acquire a broad but not an antigen-specific immunological memory (Figure 2).

Trained immunity was initially shown to act on mature myeloid cells, and this lead to the question of how this type of memory is maintained since myeloid cells have been shown to be short-lived. Recently, the issue has been resolved because trained immunity was demonstrated to occur both in bone marrow progenitor cells as well as in blood monocytes and macrophages [57,58,59].

The molecular basis by which myeloid cells are able to respond with a much more rapid and strong transcriptional responses when challenged with additional triggers has been in part defined. Evidences suggested that the trained immunity is controlled by different regulatory mechanisms which involves different players such as changes in chromatin organization, DNA methylation, expression of long non-coding RNAs (lncRNAs), and reprogramming of cellular metabolism [60,61,62,63,64]. It is important to underline that TI is considered a protective response under physiological conditions but in certain situations may cause detrimental reactions, such as those observed in auto-inflammatory diseases. Innate memory can therefore account for a possible mechanism explaining the chronic inflammatory reaction often observed in neurodegeneration, and indeed, enhanced inflammatory environment correlated with morphological changes in microglia that displayed a more reactive phenotype have been recently described [65]. Since peripheral immune training can induce memory in hematopoietic precursors in the bone marrow, such peripheral alterations may also impact not only on myeloid resident CNS cells but also on myeloid cell infiltration in the brain thus affecting neuropathology.

Peripheral inflammatory stimuli leading to long-lasting training of microglia which exacerbates CNS β-amyloidosis in a mouse model of Alzheimer disease have been demonstrated [66]. As a consequence of the epigenetic reprogramming, microglia display transcription and protein expression changes. It was shown that infections of mice very early in life seem to be able to contribute to the impairment of microglial function followed by amyloid-β-induced synapse damage and cognitive impairment by a mechanism reminiscent of trained immunity [67]. All together, these studies point out that systemic inflammation is able to induce microglia reprogramming, resulting in potentially enhanced-response with memory feature of the brain immune system. Future studies should be directed to explore this issue in human neurodegeneration.

## 5. Monocytes Migration into the Brain during Neurodegeneration

The mechanisms by which leukocytes pass through the barriers of the brain and their role in progression of neurological diseases remain yet to be fully elucidated. Although it is now accepted that the CNS undergoes immune surveillance at meningeal level [68], the mechanisms involved in immune cell trafficking in CNS remain poorly understood. The myeloid compartment in the CNS is composed of tissue-resident microglia found in the brain parenchyma and additional myeloid cells including DCs, monocytes, and granulocytes in the meningeal area.

**Figure 3:**
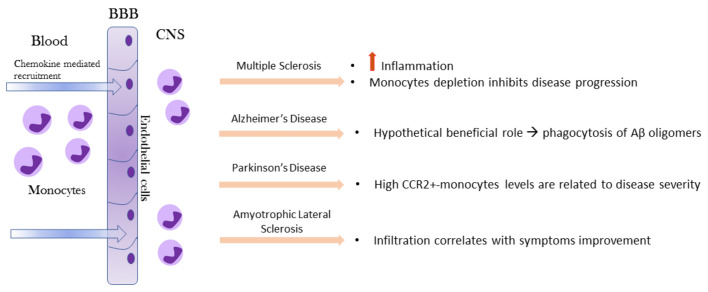
Recruitment of circulating monocytes into the brain and their impact on MS, AD, PD, and ALS. Monocytes enter the brain due to BBB disruption and in response to chemokines gradients. In the CNS compartment, monocytes assume a different role based on the specific neurodegenerative microenvironment signals. BBB, blood–brain barrier; CNS, central nervous system; MS, multiple sclerosis; AD, Alzheimer’s disease; PD, Parkinson’s disease; ALS, amyotrophic lateral sclerosis.

Under physiological conditions, monocytes are not detectable in brain or spinal cord parenchyma but only observed in the meninges [69]. Monocyte functions in the brain have been investigated primarily under pathological conditions. The recruitment of blood monocytes to the CNS following infection, injury, or an inflammatory response is often observed in neurologic disorders. After injury or during specific disease processes, the brain becomes highly permeable to circulating peripheral cells, including monocytes (Figure 3). The latter can be mobilized to cross the BBB, migrate into the brain, and subsequently contribute to the neuroimmune response in association with microglia [70]. Even though the precise mechanism is unknown, a C-C Motif Chemokine Receptor 2 (CCR2) is necessary for monocyte recruitment, through monocyte chemoattractant protein–1 (MCP-1- or CCL2) binding, expressed on monocyte surface.

A recent study conducted on human brains suggested that granulocyte–macrophage colony-stimulating factor (GM-CSF) may play a role, especially during autoimmune diseases, such as MS [71]. Compared to unstimulated cells, GM-CSF-activated monocytes were able to migrate across the BBB and to produce TNF-α, thus enhancing the inflammatory response. Beside GM-CSF and the CCL2-CCR2 axis, the CD49e (α5 integrin) was reported to play a role in monocytes brain migration. It was shown that α5 integrin is expressed only on the peripheral monocyte populations but not on CNS-resident myeloid cell populations. Treatment with α5 integrin antibody significantly reduced the experimental autoimmune encephalomyelitis (EAE) disease severity and therefore provides a strong rationale for a novel therapeutic approach that specifically targets and inhibits monocyte trafficking into the CNS thus leading to fewer deleterious side effects observed with drugs that block T lymphocytes migration [72].

Leukocyte migration to the cerebrospinal fluid (CSF) and brain is a hallmark of many pathologies of the CNS and it was found that the choroid plexus is a route of TLR2-mediated leukocyte infiltration to the CSF. Peripheral administration of the TLR2 ligand, Pam3Cys, induced marked infiltration of neutrophils and monocytes to the CSF and brain of neonatal mice. These studies suggest novel mechanisms of leukocyte migration to the brain and potential therapeutic targets to ameliorate neuroinflammation induced by meningitis or other CNS pathologies [73]. Specific inhibition of the CD40-TRAF6 axis in monocytes is also able to interfere with monocyte/macrophage transendothelial migration, but is not sufficient to strongly decrease disease severity suggesting that T cells play a major role in the EAE model. Mechanistically, the inhibition targeting CD40-TRAF6 signaling is mediated by the limitation of ROS production in monocytes and consequently a reduce migration of the cells across an in vitro BBB [74]. It remains to be established whether these pathways are operating also on the human brain.

In the EAE rodent model of MS, gene-expression profiles indicated that infiltrating monocytes are highly inflammatory compared to microglia [75]. A correlation between monocyte infiltration into the CNS and progression to the paralytic stage of the disease has been shown: depletion of monocytes was shown to significantly inhibit both disease initiation and disease progression in EAE mice [76].

In AD, monocytes are recruited at the site of Aβ deposits and in the inflammatory microenvironment around them. In fact, after migration to injured brain, monocytes can differentiate into macrophages and phagocytize protein aggregates such as Aβ [77]. MCP-1, which is produced by Aβ-induced activated microglial cells [78], triggers the mobilization of pro-inflammatory monocytes in the inflamed brain through the MCP-1 receptor CCR2 [79].

The first general evidence of immune dysregulation in PD patients was shown by measurement of elevated levels of cytokines (IL-2, IL-4, IL-6, IL-10, TNFα) in the serum and peripheral blood mononuclear cells were suspected to contribute to this peripheral cytokine elevation. For what concern monocytes brain migration, direct invasion of peripheral monocytes into the CNS has been demonstrated in an animal model for PD [80]. In humans, a strong upregulation of CCR2 on classical monocytes in Parkinson’s patients was detected whereas the percentage of these cells was specifically downregulated, suggesting that this cellular population may have migrated to the inflamed brain. Indeed, it is known that upregulation of CCR2 is essential for monocyte recruitment in inflamed tissue [81].

Similarly, in ALS, circulating human monocytes were found to be dysregulated regarding function, gene expression and subset constitution. Monocytes from ALS patients exhibited an altered adhesion capacity, which indicated a changed migratory potential. The exact role of CNS-infiltrating monocytes in ALS had remained ambiguous so far, but the mouse model of the disease (SOD1G93A tg mice) implies a role of peripheral monocytes early in the disease [82]. CNS infiltration of peripheral monocytes correlates with improved motor neuron survival in a genetic ALS mouse model [83].

Therefore, we can conclude that monocyte infiltration in brain may be both beneficial or harmful and that the exact role of these cells in different disease contexts needs further investigation. Unfortunately, we are not yet able to distinguish resident microglia from infiltrated monocytes with absolute certainty, especially in humans, and therefore we cannot rule out at the moment, the exact role of monocytes in the brain inflammatory response.

## 6. Monocytes Contribution in Multiple Sclerosis

Multiple sclerosis (MS) is an autoimmune, chronic CNS inflammatory disease leading to demyelination and neurological damage. The cause of MS is unclear but many genetic (e.g., major histocompatibility complex *HLA-DRB1* locus) and environmental factors, such as vitamin D levels, EBV infections, tobacco smoking are associated with MS [84,85]. The most frequent forms are the relapsing-remitting form (RRMS) and the primary progressive (PPMS), experienced by about 80% and 15% of MS patients, respectively [85,86]. Approximately 20–50% of RRMS progress towards the secondary progressive (SPMS) form of the disease over time. The transition from RRMS to SPMS is still not completely understood: it is thought to depend on degenerative processes in the CNS triggered by inflammation. For years, this shift was related to treatment, but a recent study demonstrates that RRMS patients without treatment are prone to develop the SPMS [87].

Active lesions characterized by prominent lymphocyte infiltration are mainly observed in RRMS, whereas a narrow rim of activated microglia and macrophages are more typically seen in PPMS lesions although other inflammatory infiltrations are present [88].

An early event in MS is the impairment of the BBB, leading to peripheral immune cell infiltration, which establishes the CNS inflammation state. In contrast to the well-defined role of T cells in MS pathophysiology, far less is known about the contribution of innate immunity [89].

Monocyte involvement in the disease was demonstrated in the EAE model where their infiltration was shown to trigger disease progression and clinical signs; the effects were abolished following monocyte and macrophage depletion [76,90,91]. It has been demonstrated that MS patient’s monocytes express high levels of metalloproteinases (MMP)-2 and MMP-14 compared to healthy controls (HCs) [92]. Because of MMP members’ strong expression, monocytes are able to migrate more rapidly across a model of the BBB in culture than T or B lymphocytes do. MMPs are the key factor for the transmigration of cells into tissues; therefore, the high migratory capacity of monocytes and their MMPs elevated expression are causally related, and indeed transmigration across an endothelial barrier is reduced when using an inhibitor of MMP activity, such as TIMP-1.

Alterations in relative distribution of monocyte subtypes were observed in MS patients and linked to disease activity, degree of disability (EDSS), and administration of disease-modifying treatment [93,94,95,96,97,98].

In general, alterations of intermediate and non-classical monocytes are associated with different inflammatory diseases [99,100,101] and in MS [94,96,97,98]. However, in MS partially conflicting results have been reported, and probably again this reflected the different clinical stages analyzed. Recently, analysis on circulating monocyte subsets has been studied in MS patients stratified by disease type course and treatment. Classical and non-classical monocyte expansion have been observed in inactive RRMS patients compared to other forms of disease and healthy controls [102]. These data clearly indicate that we strongly need to consider the specific cohort characteristic under study before drawing any general conclusion.

When examining the frequency and the phenotype of monocyte subsets in peripheral blood and cerebrospinal fluid (CSF) of RRMS, a pivotal role of CD16^+^ emerged [94]. Untreated RRMS patients have 35% less CD16^+^ in their periphery compared to HCs, whereas RRMS treated with immune-modulating drugs present the same or even higher percentage of CD16^+^ compared to HCs. The monocyte reduction in treatment-naive RRMS patients was mainly driven by non-classical monocytes (CD14^+^/CD16^++^) although the normal to high percentage in treated RRMS could be possibly attributed to direct effects of immunomodulatory drugs on the composition of the blood monocyte pool. Moreover, the naïve patients were relatively newly diagnosed, while treated RRMS have a longer disease duration. So, these monocyte perturbations could also be due to the clinical stage of the disease. Concerning CSF analysis, RRMS patients were compared to non-inflammatory neurological disorder (NIND) patients. The cytometric analysis revealed that the percentage of CD16^+^ monocytes was reduced in CSF of RRMS patients suggesting that they may have migrated in the meninges and in the parenchyma area.

In the light of their CD16^+^ data, Waschbisch et al. suggested that the decrease in CSF monocytes is mainly driven by a reduction in the CD16^+^ monocyte subset. This is due to a higher propensity of CD16^+^ monocytes to adhere to adjacent tissue and turn into monocyte-derived subarachnoid-space macrophages compared to that of classical monocytes. Beside CD16^+^ cells facilitate CD4^+^ T cells migration, which is a typical mechanism present in MS pathology. Unfortunately, the study did not analyze the HCs CSF due to difficulties in obtaining these sample types in HCs. Additional insights on human MS cellular subsets composition are emerging from single cell analysis. Single cells transcriptomics of blood and CSF fluid from MS patients and controls lead to identification of unknown myeloid dendritic cell populations (mDC), of a CD4^+^ T cells expansion with cytotoxic phenotype and of a late-stage B cell lineage in the CSF in MS [103]. It remains to be established whether these recent findings are correlated to the specific cohort analyzed or they are a general feature of the disease. We anticipate it to be related to a specific clinical stage of the MS disease analyzed.

Recently, monocyte microRNA (miRNA) analysis between RRMS and PPMS has been described [104]. Twenty-one RRMS patients (6M/15F, mean age 38 ± 9, EDSS 2.9 ± 1.4) and eight PPMS patients (1M/7F, mean age 47 ± 11, EDSS 5.9 ± 1.3) and 16 HCs (10M/6F, mean age 45 ± 11) were studied. MiRNAs with anti-inflammatory functions, which promote pro-regenerative polarization, were increased in MS patients, while the pro-inflammatory miR-155 was downregulated in the same patients. These changes may reflect the attempt of monocytes to establish an anti-inflammatory/pro-regenerative response in MS. This is in line with the clinical status of the enrolled MS patients. However, miR-124, another anti-inflammatory miRNA, was strongly downregulated, especially in PPMS, suggesting persistent monocyte activation during disease progression.

Finally, the role of monocyte subsets in MS was investigated in the mouse model of MS, by using single-cell analysis [69]. In this study, six different monocyte subtypes, four of which were previously unknown, were identified. Interestingly, the author’s group depleted the population with antibodies against CCR2 and as expected, the cells died and the MS symptoms in the mice decreased within a short period of time. Nevertheless, further analysis showed that only monocytes expressing Cxcl10 were destroyed by the antibody treatment concluding that the Cxcl10^+^ cells were primarily responsible for causing MS tissue damage in the brain. In addition, it was shown that the Cxcl10 monocytes attract T cells and produce large amounts of interleukin-1-beta (IL-1β), a cytokine able to open the BBB, enabling immune cells to more easily pass from the blood to the brain and exacerbate the symptoms. Therefore, specifically eliminating the Cxcl10^+^ monocytes instead of targeting the T or B cells of the immune system could be a strategy as this would protect the body’s immune memory and prevent many side effects of the current MS therapies. However, to translate these findings into a clinical setting, there is a need to demonstrate that the Cxcl10^+^ monocytes subset also exists in humans [69].

All these recent findings emphasize the role that monocytes play in MS disease elucidating the role of innate immunity in MS.

## 7. Monocytes in Alzheimer’s Disease

Alzheimer’s disease (AD) is the most common dementing neurodegenerative disorders that it is characterized by two hallmarks: extracellular deposition of β-amyloid plaque (Aβ) and intracellular neurofibrillary tangles (NFTs) made up of the hyperphosphorylated microtubule-associated τ. This toxic aggregation determines cognitive decline and death. Aβ starts with a sequential cleavage of amyloid-b-protein precursor (APP), by β and γ secretases to produce insoluble Aβ fibrils [105]. Then Aβ oligomerizes causing toxic aggregation. This polymerization induces kinase activation, leading to hyperphosphorylation of the microtubule-associated t protein, which polymerizes in turn forming insoluble NFTs. Indeed, it has been demonstrated that soluble Aβ controls τ phosphorylation [106].

Although the majority of AD cases are sporadic, there are also familiar forms, caused by three principal mutations in: amyloid precursor protein (APP), presenilin 1 (PSEN1), and presenilin 2 (PSEN2) [107]. Single nucleotide polymorphisms in several other genes have recently been shown to be associated with increased or decreased risk for developing late-onset AD. The common APOE ε4 allele explains a substantial part of, but does not completely account for the heritability of AD. Genome-wide association studies have identified more than 30 genetic loci for AD, many of them were shown to be related to the immune response and microglia [108]. Among them, CD33 and TREM2 mutations were identified to be associated with an increased risk of developing AD [109,110]. While microglia is important to clear amyloid beta (Aβ), it can also release pro-inflammatory cytokines increasing neuroinflammation [111]. Therefore, the understanding of the mechanism that controls myeloid cells in the brain could advance therapies for AD.

Several studies have demonstrated a close relationship between neuroinflammation, and AD pathology and inflammatory components have been identified in AD lesions [112]. The neuroinflammatory reaction has been exclusively linked to Aβ [113]. In AD, as well as in neurodegenerative diseases in general, the damage is associated with an increase in the BBB permeability, which favor peripheral cell CNS infiltration. This mechanism is mediated by cytokines and chemokines, which may attract peripheral cells such as monocytes [114]. The BBB model was formed by a monolayer of human endothelial cells derived from cerebral micro vessels and human astrocytes separating the vascular side (upper chamber) from the brain parenchymal side (lower chamber) [115,116].

Through these experiments, it was demonstrated that Aβ_1-42_ had effects on circulating monocytes in a dose and time-dependent manner. Addition of Aβ_1-42_ in the lower chamber resulted in a huge increase in transmigration of monocytes after 24 h compared to controls suggesting that indeed, Aβ_1-42_ attracts peripheral monocytes. In addition, the presence of both Aβ_1-42_ and a small number of monocytes in the lower chamber further increases the transmigrated monocytes as opposed to Aβ_1-42_ only.

It is known that Aβ induces chemokine release such as MCPs, which can attract monocytes, and that in turn they start to produce proinflammatory cytokines like TNF-a and IL-6. The activated macrophages are known to improve their phagocytic capacity of toxic elements, including Aβ [77]. It is reasonable to think that some of the reactive microglia-like cells surrounding the amyloid plaque cores may also be derived from peripheral monocytes/macrophages.

Numerous studies have shown the capacity of Aβ to invoke the secretion of proinflammatory factors by monocytic cells. In AD, the BBB disruption has been proposed as a co-cause of sporadic AD besides other mechanisms involved in the dementia progression [117,118]. Indeed, patrolling monocyte subset (non-classical monocytes) adhered to Aβ-rich brain vasculature in a specific way, eliminating Aβ aggregates and transporting them to the blood circulation [119].

Pro-inflammatory monocytes have been shown to infiltrate the brain and differentiate into activated macrophages. Non-classical monocytes (CD14^+^/CD16^++^) are reduced in AD patients compared to mild cognitive impairment patients or healthy controls suggesting that this monocyte subset may have a protective role in the disease [120]. On the contrary, non-classical monocyte depletion has been shown to improve the disease in the mouse model of AD indicating that there may be a difference in mice than in humans [121]. However, in a different patient’s cohort, a progressive reduction of classical monocytes was observed [122]. In particular, this reduction was mainly observed in the mild and moderate/severe form of AD dementia suggesting a more prominent role in this disease’s clinical stages for classical monocytes. At the same time, a redistribution of monocytes leading to an increase of intermediate and non-classical monocytes emerged from the same study indicating that a dysregulation of the monocyte subset distribution may participate in the disease process [122]. It remains to be clarified whether there is a shift of the monocyte phenotype or there is a progressive death of classical monocytes.

Finally, it has been shown that aging is also an important factor for AD development [123] and recently data indicated that monocyte Aβ uptake decreases with age especially in the AD population implying that compromised Aβ uptake by monocytes is involved in AD pathogenesis [124,125,126]. The different monocyte subsets may have different functions in AD, and indeed, the intermediate subset is highly phagocytic compared to classical and non-classical monocytes, nevertheless, Aβ uptake ability was decreased in all subsets in AD patients. In addition, the intermediate subset was shown to release less IL10 than usual indicating that the mechanisms underlying the alteration in Aβ uptake ability by monocytes in AD patients are different from those associated with aging [127]. Therefore, the issue remains to be further investigated, and it suggests that the recovery of Aβ uptake function by blood monocytes could be of therapeutic value for AD. Cellular and molecular therapies able to modify monocyte functions should also be considered in the future of AD therapeutic development.

## 8. Alteration of Monocytes in Parkinson’s Disease

Parkinson’s disease is a neurodegenerative movement disorder characterized by a progressive loss of dopaminergic neurons in the substantia nigra pars compacta and accumulation of misfolded α-synuclein, which is the major constituent of fibrillary aggregates called Lewy bodies (LBs) [128]. As for the other neurodegenerative disorders, the etiology is unknown. Most cases of Parkinson’s are classified as sporadic, while approximately 10% of people with PD have the familiar form. These genetic variants can be caused by mutations in a set of genes, such as Parkinsonism Associated Deglycase (PARK7), which play a role in oxidative stress, and PTEN Induced Kinase 1 (PINK1) and Parkin RBR E3 Ubiquitin Protein Ligase (PRKN), which regulate mitochondrial functions, and alpha synuclein (SNCA) [128]. During the last years, a correlation between innate immune system and Parkinson’s disease emerged, with a special consideration for the role of monocytes.

Monocytes were suggested to be a contributing factor to PD pathogenesis based on a study in which an over-representation of expression quantitative trait loci (eQTL) specific to monocytes was linked to PD [129]. Several studies demonstrated an enrichment of classical monocytes in peripheral blood of PD patients, especially in those with a high risk of developing early dementia (HR-PD), based on neuropsychological predictors genotype [130,131,132]. Moreover, monocytes of HR-PD patients express higher levels of triggering receptor expressed on myeloid cells 2 (TREM2), a critical regulator of inflammation [131].

Phenotypic analysis has revealed that classical monocytes expressing CCR2 are enriched in the blood of PD patients and that at the same time a strong reduction of CCR2-positive cells in peripheral blood was reported [81,133]. A possible explanation is that classical monocyte CCR2^+^ are attracted to the inflamed brain of PD patients, since dopaminergic neurons are a source of CCL2 release in PD mouse model [134]. Moreover, a link between CCR2^+^ monocytes and disease duration was observed, confirming that the activation of CCL2-CCR2 axis plays an important role in PD. Another interesting finding is that the blood of PD and HR-PD patients presents a higher production of the monocytic precursors leading to increased monocyte production, and confirming previous observations about monocyte enrichment in the brain of PD patients [81]. When stimulated with a pro-inflammatory stimulus such as LPS, PD monocytes show an excessive inflammatory profile with upregulation of IL-1β, IL-6, IL-8, and IFNγ and an abnormal CCL2 expression [130]. More interestingly, these processes are related to PD severity. Nevertheless, data reported by Grozdanov, V. et al. were in disagreement with other studies [135,136], suggesting once again, that different ways of monocyte isolation and different cohorts of patients may account for the discrepancy. In addition, the same study also revealed that the phagocytic activity of PD monocytes was downregulated when cultivated in standard medium and not in the presence of autologous serum [131]. This suggested that extrinsic cellular components can influence monocyte functionality, and this issue should be taken into account when comparing different studies.

Another remarkable aspect is regarding monocyte activity in PD and a protein involved in depression. After some years from the diagnosis, many PD patients experience different forms of depression, and indeed, levels of the P11 (S100A10) protein, involved in major depressive disorder, were shown to be expressed almost 10-fold higher in monocytes than in the other leukocytes in PD-depressed patients [137]. Interestingly, PD patients without depression, and therefore in the early phase of the disease, did not present the same high levels. So, it could be concluded that the protein p11 could be a possible biomarker for monitoring the severity of PD, especially in those patients in which comorbidity with depression is present [137].

Finally, PD monocyte transcriptomes were studied in an effort to identify blood-based biomarkers in PD [138,139,140]. Analysis of the transcriptomic signature in monocytes from PD patients in their early disease course was also defined [32]. In this study, human monocyte transcriptomes from 10 male healthy individuals were compared with monocytes isolated from male individuals in the early clinical stage of PD by RNAseq analysis. A distinct signature that separates PD and controls based on clinical score and disease duration was isolated. Genes belonging to the functional classes of leukocyte migration and regulation of immune responses were enriched, suggesting the link between innate and adaptive immune responses. This indicated monocytes as a potential cell to study at different disease stages in PD patients to decipher the time course of the neuroinflammatory response. Future studies are needed to directly compare monocyte populations with different functions to define specific inflammatory signature. Recently, the soluble CD163 (sCD163) molecule, a well-known protein released by the monocyte cell lineage, but not by microglia, lymphocytes, or neurons, was suggested to serve as a disease biomarker [141]. Since sCD163 is constitutively produced in serum and CSF upon immune signals, it is suggested to be used as an early and late PD biomarker to evaluate monocytic activation in different PD stages. However, the study needs to be extended to a large cohort of PD patients and healthy individuals to validate and reinforce these findings.

## 9. Monocytes Plasticity in Amyotrophic Lateral Sclerosis

Amyotrophic lateral sclerosis is a debilitating neurodegenerative disease with reported immune dysregulations [83,142,143,144]. Different studies have reported that peripheral immune system cells are functionally altered, especially those with myeloid lineage [145,146,147,148,149]. One of the most challenging factors in most neurodegenerative disease, including ALS, is their heterogeneity of clinical features which render it challenging to identify factors that alone may explain all the pathological mechanisms that eventually are operating in the disease. Any given cohort of patients varies in terms of severity, progression, site of onset, degree of respiratory involvement, and degree of upper or lower motor neuron involvement [150].

Within the myeloid population so far studied, monocytes have been reported to play a role [147]. ALS patients with distinct clinical features have differential monocyte cell subset distribution, for example patients with greater disease severity, as determined by a lower revised amyotrophic lateral sclerosis functional rating scale score, showed a reduced non-classical monocyte subset whereas patients with greater bulbar involvement had a reduction in the proportion of classical, intermediate, and non-classical monocyte populations. On the same line, CD16 expression in neutrophils increased in patients with greater disease severity and a faster rate of disease progression, whereas HLA-DR expression in all monocyte populations was elevated in patients with greater respiratory impairment [151].

Previous literature reporting on immune cell frequencies and marker expression has not revealed consistent findings again probably because of the heterogeneity of patients and methodological variations between studies. We should always keep in mind that each cohort population under study is unique and therefore what we observe in one cohort may not be valid for others. To avoid confusing factors, guidelines on immunophenotyping in whole blood should be adopted to be able to compare results from different studies [152,153].

ALS monocytes skewing toward a proinflammatory state have been investigated by RNAseq analysis. Gene expression profiles were studied in 23 ALS monocytes compared to 10 healthy control individuals, and demonstrated that monocytes isolated from patients with ALS expressed a unique gene profile associated with proinflammatory immune responses. The most upregulated genes (9 out of 10) were associated with the pro-inflammatory monocytes’ response, such as IL-1β and IL-8 [149]. These findings were validated through qRT-PCR in an additional cohort confirming the higher mRNA expression values in monocytes of ALS patients. Furthermore, CXCL1, CXCL2, and NLRP3 were upregulated in ALS monocytes [149]. These results were obtained from monocytes isolated by negative selection, which could have a lower impact on cellular activation compared to positive selection. Similar findings were reported by performing RNA-seq on CD14^++^ from peripheral blood of five ALS patients and eight HCs, which revealed 420 DEGs (FC ± 1.5, FDR ≤ 0.05) in which inflammatory genes, such as ICAM-1, IL-8, CCR1, and JUN were profiled. These results strongly indicated monocytes of patients with ALS to be associated with disease pathogenesis [148].

ALS peripheral monocytes produce more pro-inflammatory cytokines when stimulated with LPS and IFNγ to differentiate into M1 phenotype suggesting that ALS monocytes are functionally altered, which could explain an increased cytotoxicity once they arrive into the CNS [154]. The functional alteration in ALS monocytes was also demonstrated by examining the adhesion capacity of ALS monocytes, which suggested a change in their migratory capacity. Indeed, the number of adhering monocytes is higher in ALS than HCs after LPS stimulation, and monocyte transmigration is known to be preceded by extravasation and adherence to the vessel walls [148].

Further supporting the role of monocytes in ALS pathogenesis is the finding that the transactive response DNA-binding protein 43 (TDP-43) is accumulated in a subgroup of ALS cases again underlying the possibility that different mechanisms of disease are operating in different cohorts of patients [155]. These issues should be further deeply investigated as we hypothesize that they will explain the great clinical heterogeneity we observe in ALS and in general in neurodegenerative diseases.

Finally, it will be important to clearly distinguish microglia from peripheral blood-derived monocytes infiltrating the brain. Recently, the CD169/Siglec-1 molecule was suggested as a marker for monocytes in the CNS, because it is not expressed in resident microglia [147,156]. By using this molecule, it was possible to show that CD169^+^ cells were significantly higher in lumbar spinal cords of 10 ALS patients. The ALS CD169^+^ monocytes were shown to have a decreased diameter, and to be located within the tissue (80.2%) with only a small percentage within the perivascular space (19.8%) [148]. This finding might further be correlated with a different stage of monocyte activation.

Interestingly, in SOD^G93A^ ALS mouse model, immunomodulatory treatment increased the CD169^+^ cells that correlated with the enhancement of motor neuron survival, suggesting that monocyte invasion at least in this experimental model, acted as a neuroprotective in the early stage studied.

In conclusion, monocytes may have a role in ALS pathogenesis therefore, it can be hypothesized that suppression of their pro-inflammatory phenotype may provide a new therapeutic option for ALS. Nevertheless, to reach this end point, there is a need to further expand our knowledge at the monocyte single cell level to precisely identify the specific monocyte subset infiltrating the brain that may exert a pathogenic as well as protective effect in this disease.

## 10. Conclusions

For a long time, the brain was considered an immune-privileged organ, thus neglecting the possibility that peripheral cells impact neurological and neurodegenerative diseases. Thanks to the most recent discoveries, the role of the immune system has been increasingly at the center of new and interesting areas of research pointing to the peripheral cell–brain interconnections. Although the major function of monocytes is to provide defenses against infection and injury, their impacts on brain function have been increasingly recognized. Under pathological conditions, monocytes may permeate the BBB, differentiate in macrophages and modulate neuronal function by releasing inflammatory mediators. In this review we analyze the possible role of peripheral monocytes in four of the most common neurodegenerative diseases: MS, AD, PD, and ALS. What emerged is that the results vary according to the cohort of patients analyzed and the severity and the clinical stage at which the disease has been studied. The role of monocytes have been better characterized in MS, whereas their precise contribution to AD, PD, and ALS have yet to be fully revealed in part due to the difficulty of distinguishing these cell type both morphologically and functionally from the resident microglial cells.

The complicated mix to consider includes monocyte redistribution, phenotypic changes, cytokine secretion, and functional changes of these cell types. In addition, the microenvironment in which the cells interact is also very important and probably is going to play a major role in determining the outcome of the exact cellular phenotype and function in each specific organ.

Activation of the CNS innate immunity is now recognized to be a characteristic of neurodegenerative and chronic disorders. Microglia and infiltrating monocytes participate in shaping the neuroinflammatory microenvironment, and now different studies have demonstrated that these myeloid cell populations can orchestrate different aspects of CNS inflammatory responses. Myeloid cells can either protect or exacerbate CNS disease, based on the context of specific pathological mechanism and etiology. Therefore, reliable models that study myeloid cells with the contribution of its microenvironment will be instrumental to identify novel immune-modulating and repairing strategies for CNS-related inflammatory disorders.

Future studies focusing on single cells and other more sophisticated technologies such as brain organoids will help us to address these and other conflicting issues in the near future. The understanding of the heterogeneity and functions of monocyte subsets in both homeostasis and disease will allow the development for new and better therapeutic approaches that will selectively target monocyte populations instead of targeting all monocytes as a whole.

Finally, it should be emphasized that one of the greatest difficulties lies in trying to understand this connection by studying human samples. Much still needs to be explored, but certainly monocytes besides other myeloid component, as well as the immune system in general and the activity within each organ, are no longer a separate thing, but play a central role in the development of neurodegenerative diseases. It will be important to understand more deeply what molecular mechanisms underlie this involvement, to look for new drugs or therapies that target monocytes subsets, and not just nerve cells.

## Figures and Tables

**Table 1:** Monocytes subsets and their main functions. Human monocytes are classified as classical (CD14^++^/CD16^−^), intermediate (CD14^++^/CD16^+^) and nonclassical (CD14^+^/CD16^++^) monocytes.

Human Monocytes Subsets	Percentage	Molecular Markers	Additional Molecular Markers	Main Role
Classical	85–90% of the total circulating monocytes	CD14^++^/CD16^−^	Low levels of TLRs High levels of CD80, CD86	Phagocytosis and immune response
Intermediate	The remaining 10–15%	CD14^++^/CD16^−^	High levels of TLRs 2, 4, 5 CD80, CD86, HLA-DR	Proinflammatory function and wound healing
Non Classical		CD14^+^/CD16^++^	High levels of CD80, CD86	Antigen presentation and patrolling role

## Data Availability

Not applicable.
